# Supplementary material for: Prevalence of Sleep Disorders, Risk Factors and Sleep Treatment Needs of Adolescents and Young Adult Childhood Cancer Patients in Follow-Up after Treatment
Source: Cancers (Basel). 2022 Feb 13;14(4):926. doi: 10.3390/cancers14040926 (PMC8870170; doi:10.3390/cancers14040926)
Supplement: Supplementary file 1 [file cancers-14-00926-s001.zip › cancers-1584664-supplementary.pdf]

Supplementary Material

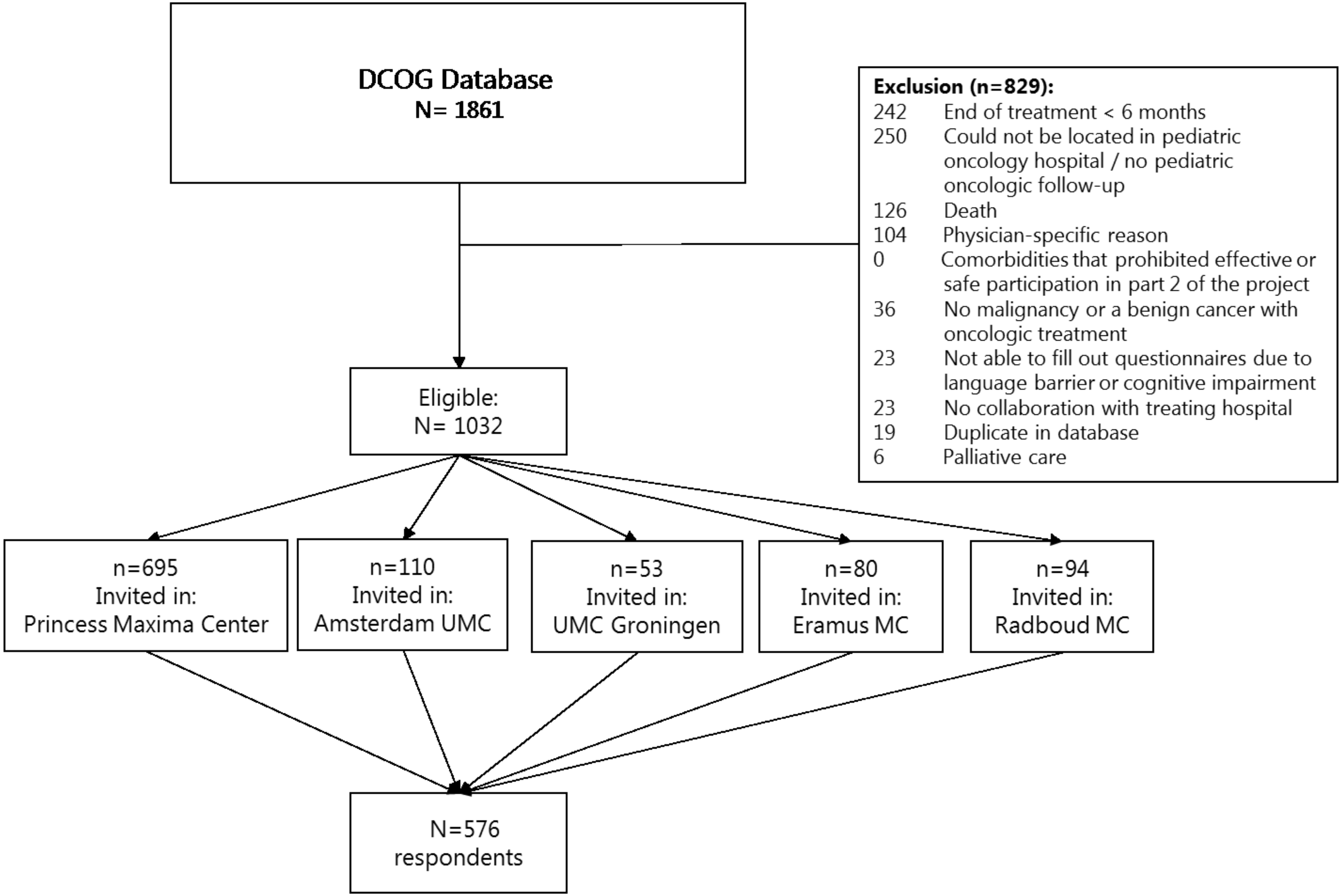

Figure S1. Flow chart of patient inclusion

Table S1. Sleep medication use per sleep disorder

n (%)	RLS-LMS (n=43)	SBD (n=10)	Parasomnia (n=20)	Hypersomnia (n=20)	CRSD (n=46)	Insomnia (n=54)	No sleep disorders (n=468)
Use homeopathic remedies	4 (9.3)	1 (10.0)	1 (5.0)	1 (5.0)	0 (0.0)	2 (3.7)	2 (0.4)
Use benzodiapizine	1 (2.3)	0 (0.0)	0 (0.0)	0 (0.0)	0 (0.0)	0 (0.0)	5 (1.1)
Use melatonin	7 (16.3)	3 (30.0)	5 (25.0)	5 (25.0)	8 (17.4)	11 (20.3)	32 (6.8)
Used sleep medication in the past month	13 (30.2)	5 (50.0)	6 (30.0)	6 (30.0)	8 (17.4)	14 (25.9)	38 (8.2)

Note. Patients that use multiple medications count in multiple cells.

Table S2. Rates pre- and during the covid-19 pandemic.

	**Total group (N=565)**			**Adolescents (n=323)**		**Young adults (n=242)**	
	**Pre-covid-19 pandemic, n (%) n=367**	**During covid-19 pandemic, n (%) n=198**	OR (95%CI)[1]	**Pre-covid-19 pandemic, n (%) n=198**	**During covid-19 pandemic, n (%) n=125**	**Pre-covid-19 pandemic, n (%) n=169**	**During covid-19 pandemic, n (%) n=73**
Insomnia	44 (12.0)	10 (5.1)	0.41 (.20-.83)*	18 (9.1)	6 (4.8)	26 (15.4)	4 (5.5)
CRSD	38 (10.4)	8 (4.0)	-	12 (6.1)	6 (4.8)	26 (15.4)	2 (2.7)
RLS-LMS	34 (9.2)	9 (4.5)	-	13 (6.6)	8 (6.4)	21 (12.4)	1 (1.4)
Parasomnia	16 (4.4)	4 (2.0)	-	5 (2.5)	4 (3.2)	11 (6.5)	0 (0.0)
Hypersomnia	17 (4.6)	3 (1.5)	-	4 (2.0)	2 (1.6)	13 (7.7)	1 (1.4)
SBD	8 (2.2)	2 (1.0)	-	2 (1.0)	1 (0.8)	6 (3.6)	1 (1.4)

Note. OR and 95% CI are presented, if significant asterisks were added * <.05, **<.01 ***<.001. 1. Analysis is adjusted for age. Only those with a sample ≥10 per event are tested.

Post-hoc analyses: differences between pre versus post covid-19 pandemic recruitment

Patients recruited pre the covid-19 pandemic (Nov-18 until Feb-20) showed higher prevalence rates of sleep disorders compared to those recruited during the covid-19 pandemic (March-20-July-21), see Supplement 3. Since samples were small, only insomnia rate could be statistically evaluated, which was significantly decreased during the covid-19 pandemic (OR 0.41, 95% CI .20-.83, p <.05) controlled for age. When assessing sample differences, the sample recruited post-pandemic was younger (pre M = 17.2 SD =2.84 versus post M = 16.6, SD =2.98) and contained less young adults (pre 46.0% versus post 36.9%, OR 0.70, 95%CI .50-1.00, p =.05). Furthermore, there were no differences in sex, educational level and co-morbid health conditions (p >.05).
